# Job burnout among primary healthcare workers during COVID-19 pandemic: cross-sectional study in China

**DOI:** 10.3389/fpubh.2023.1266864

**Published:** 2023-12-06

**Authors:** Xianming Cai, Tianshuo Zhao, Linyi Chen, Sihui Zhang, Ailing Yu, Xihong Sun, Shengnan Gao, Yuanshan Zhang, Chao Wang, Juan Du, Yaqiong Liu, Qing-Bin Lu, Fuqiang Cui

**Affiliations:** ^1^Department of Epidemiology and Biostatistics, School of Public Health, Peking University, Beijing, China; ^2^Department of Laboratorial Science and Technology, School of Public Health, Peking University, Beijing, China; ^3^Center for Infectious Diseases and Policy Research and Global Health and Infectious Diseases Group, Peking University, Beijing, China; ^4^Key Laboratory of Epidemiology of Major Diseases (Peking University), Ministry of Education, Beijing, China; ^5^Department of Epidemiology, University of Pittsburgh, Pennsylvania, PA, United States; ^6^Gansu Provincial Center for Disease Control and Prevention, Lanzhou, Gansu, China; ^7^Jining Center for Disease Control and Prevention, Jining, Shandong, China; ^8^Harbin Nangang Center for Disease Control and Prevention, Harbin, Heilongjiang, China; ^9^Dezhou Lingcheng Center for Disease Control and Prevention, Dezhou, Shandong, China

**Keywords:** job burnout, COVID-19, primary healthcare workers, occupational health, MBI scale

## Abstract

**Objective:**

This study evaluated job burnout among primary healthcare workers (PHCWs) in China during the COVID-19 pandemic, explored its influencing factors, and examined PHCWs' preferences for reducing job burnout.

**Method:**

We conducted a multicenter cross-sectional study in Heilongjiang, Sichuan, Anhui, Gansu, and Shandong Provinces. An electronic questionnaire survey was conducted through convenience sampling in communities from May to July 2022. We collected sociodemographic characteristics, job burnout level, job satisfaction, and preferred ways to reduce job burnout among PHCWs.

**Results:**

The job burnout rate among PHCWs in China was 59.87% (937/1565). Scores for each dimension of job burnout were lower among PHCWs who had a better work environment (emotional exhaustion OR: 0.60; depersonalization OR: 0.73; personal accomplishment OR: 0.76) and higher professional pride (emotional exhaustion OR: 0.63; depersonalization OR: 0.70; personal accomplishment OR: 0.44). PHCWs with higher work intensity (emotional exhaustion OR: 2.37; depersonalization OR: 1.34; personal accomplishment OR: 1.19) had higher scores in all job burnout dimensions. Improving work environments and raising salaries were the preferred ways for PHCWs to reduce job burnout.

**Conclusion:**

Strategies should be developed to improve job satisfaction among PHCWs, enhance their professional identity, and alleviate burnout to ensure the effective operation of the healthcare system, especially during periods of overwork.

## 1 Introduction

Job burnout is an important issue in the field of occupational health. A response to prolonged exposure to workplace stress, burnout is a syndrome manifested by emotional exhaustion, depersonalization, and a diminished sense of personal accomplishment at work (1). Job burnout has three main characteristics: (1) a feeling of energy expenditure or exhaustion, (2) increased perceptual distance from work or negative work-related emotions or feelings of cynicism, and (3) lowered professional performance. Burnout can occur in various industries and can be costly, resulting in employee tardiness, absenteeism, turnover, decreased performance, or even negative employee health outcomes (2–5).

Studies in Europe and the US have shown that long work hours are a major cause of burnout (6, 7). The phenomenon of long work hours is commonly found among healthcare workers worldwide, and the situation is particularly critical in China (1). The long, high-intensity work hours characteristic of healthcare work cause these workers to be highly prone to burnout. Job satisfaction is defined as the extent that the health workers are positive, negative or affective toward their work (8). The 2011 China Primary Care Workforce Survey showed that low job satisfaction and high occupational burnout were widespread (9). It was confirmed that lower job satisfaction can significantly contribute to job burnout of healthcare workers (10, 11). Studies suggest that healthcare worker burnout has both direct and indirect negative effects on healthcare institutions, healthcare workers themselves, and patients, including errors in diagnosis and treatment (12, 13), lowered professionalism and efficiency in healthcare services (14), and risks to the health and safety of physicians (15, 16). Burnout can even affect the orderly functioning of the whole healthcare system (14). Compared with the West, Asia has limited research on healthcare worker burnout, although its overall level is quite high. Cross-sectional studies in Malaysia (17), Yemen (18), and Hong Kong (19) found that more than 30% of healthcare workers had a high degree of job burnout. The Maslach Burnout Inventory (MBI) is the most widely used scale for measuring job burnout, includes subscales purported to measure each of these three dimensions. Many researchers have found the MBI had the greatest predictive validity (20).

China has a large population (21) and 2.2 physicians per 1,000 people (22), which is below the World Health Organization's recommendation. As a result, Chinese healthcare workers generally work long hours and have heavy workloads. High levels of burnout are prevalent among China's healthcare workers. One systematic review estimated that the job burnout rate in the medical field in China was 66.5–76.9% (23). A national cross-sectional survey of physicians in Chinese tertiary hospitals found that 38.4% of respondents met the criteria for burnout (24).

Since 2019, the COVID-19 pandemic has brought new challenges to healthcare workers. Primary healthcare workers (PHCWs) in China have made great contributions and borne high work stress under the country's evolving pandemic-prevention policies. It is important, then, to assess the level of burnout among PHCWs in China during the COVID-19 pandemic, analyze the factors affecting burnout, and explore PHCWs' preferences for reducing job burnout to promote healthy career development. To this end, we conducted a cross-sectional study to assess the prevalence of burnout among PHCWs during the COVID-19 pandemic and explore the factors affecting burnout.

## 2 Methods

### 2.1 Data collection

We used a cross-sectional survey method and selected five provinces (Heilongjiang, Sichuan, Anhui, Gansu, and Shandong) as survey sites to recruit subjects from May to July 2022. Using nonrandom convenience sampling, we recruited subjects from the community who met the survey criteria. The selected study subjects filled out an anonymous questionnaire via an online platform (Survey Star, Changsha Ran Xing Science and Technology, Shanghai, China). The key variables in the questionnaire were all required and assigned logical values. Data were screened according to the requirements of the study, finally the questionnaire information of 1,561 cases were selected, and then the database was locked.

### 2.2 Study subjects

The inclusion criteria for survey respondents included the following: they needed to be PHCWs who had online access so they could complete the survey. Participation was voluntary.

Sample size calculation was based on the cross-sectional survey design. The overall burnout indicator for healthcare work obtained from the data was approximately *p* = 0.3, α = 0.05, and *d* = 0.1 × *p* = 0.03. The sample size for a purely random sample was derived from the formula for cross-sectional survey sample size. Considering the sample size expansion (1.5–2.0 times) problem for nonrandom sampling, the minimum sample size is expanded to *N*_*srs*_ = 897 × 1.5 = 1,346:


$$\begin{array}{l} N_{srs}=\frac{t_{\frac{\alpha }{2}}^{2}\times P\left(1-P\right)}{d^{2}}. \end{array}$$

### 2.3 Measures and variables

The questionnaire was divided into four parts:

1. Basic sociodemographic characteristics, such as gender, technical title, work unit, years of work, and education level.
2. Maslach Burnout Inventory (MBI): The MBI contains three dimensions: emotional exhaustion, depersonalization, and lack of personal accomplishment. The scale was designed by Maslach and Jackson (25) and was adapted and refined for China by ChaoPing Li of Renmin University of China.
3. Job satisfaction, divided into three evaluation aspects: work environment, salary, and work intensity.
4. Preferences for reducing job burnout: Five improvement methods are given: (1) awarding honorary certificates or titles, (2) reducing work intensity, (3) improving work environment, (4) providing opportunities for further education, and (5) increasing salaries. The survey of preference for improving job burnout adopts the method of option ranking. PHCWs were first asked to select the three options that they personally thought would be most effective in improving burnout, and then the three options were ranked from most important to least important.

### 2.4 Burnout definition

Burnout was measured using the MBI scale, quantified using the Likert-type scale, and evaluated according to the SS′ scoring principle: SS′ = 0.4 × mean score for emotional exhaustion + 0.3 × mean score for depersonalization + 0.3 × (6 – mean score for personal accomplishment) (26, 27). Based on the scores, the subjects were divided into three categories: (1) no job burnout (0 ≤ SS′ < 1.50), (2) mild job burnout (1.50 ≤ SS′ < 3.50), and (3) severe job burnout (3.50 ≤ SS′ < 6).

In this study, mild and severe job burnout are regarded as the levels of job burnout that are in need of improvement; that is, the detection rate of job burnout is positive:


$$\begin{array}{l} Job\;burnout\;rate=\;\frac{mild\;job\;burnout+severe\;job\;burnout}{total\;number}\times 100\%. \end{array}$$

### 2.5 Statistical analysis

The questionnaire was analyzed using R 4.1.2 (R Development Core Team) and IBM SPSS AMOS 26.0.0 (IBM Corporation, Armonk, NY, USA). Differences were statistically significant at *p* < 0.05.

We used the chi-square test to analyze the correlation between the job burnout level of healthcare workers and demographic factors. Stepwise logistic regression was used to analyze the factors affecting job burnout. We established a structural equation model (SEM) based on theoretical assumptions and the factors affecting burnout to explore the path coefficients of potential variables influencing burnout. The generalized least-squares (GLS) method was used to estimate the path coefficients. We computed the fit of the model to the data using the following: chi-squared/degree of freedom (CMIN/df), root-mean-square error of approximation (RMSEA), goodness-of-fit index (GFI), adjusted goodness-of-fit index (AGFI), and comparative fit index (CFI). Furthermore, multigroup SEM was used to explore similarities and differences in the model according to age, gender, years of work, and whether engaged in new COVID-19-related work.

We used Thurstone's pairwise comparison method to analyze the ranked items of burnout improvement methods. In this method, option combination information is converted into pairwise comparison information, and the value of column *j* for row *i* is divided into three cases: *R*_α_, *R*_β_, and *R*_γ_. The formula for calculating the probability table *p*_*ij*_ and the scale value *S*_*i*_ is


$$\begin{array}{l} p_{ij}=\frac{R_{\alpha }+R_{\beta }+0.5\times R_{\gamma }}{N},\;\\ S_{i}=\frac{\sqrt{2}}{n}\overset{n}{\underset{j=1}{\sum }}x_{ij}\;\left(i=1,\;2,\;\ldots ,\;n\right). \end{array}$$

## 3 Results

We collected 1,561 valid questionnaires. Among the investigated PHCWs, the average age was 37.50 ± 10.30 years, 1,139 (72.97%) were female, and 45.16% (705/1,561) worked in rural areas. Most worked in village clinics (45.16%) and community healthcare centers (41.13%). Table 1 shows the personal and professional characteristics of the respondents.

**Table 1:** Social demographic and burnout level of PHCWs.

**Characteristics**	**Total (%)**	**Without job burnout (%)**	**With job burnout (%)**	***P***
	***N*** = **1,561**	***n*** = **628**	***n*** = **933**	
**Sex**				**0.310**
Male	422 (27.03)	179 (28.50)	243 (26.05)	
Female	1,139 (72.97)	449 (71.50)	690 (73.95)	
**Age**				<**0.001**
≤ 35 years	737 (47.21)	245 (39.01)	492 (52.73)	
>35 years	824 (52.79)	383 (60.99)	441 (47.27)	
**Work unit**				**0.037**
Center for Disease Control and Prevention	123 (7.88)	54 (8.60)	69 (7.40)	
Grade III Level A hospital	91 (5.83)	37 (5.89)	54 (5.79)	
Community healthcare center	642 (41.13)	231 (36.78)	411 (44.05)	
Township Health Center and Village Clinic	705 (45.16)	306 (48.73)	399 (42.77)	
**Technical title**				>**0.999**
Junior or unverified	1,018 (65.21)	410 (65.29)	608 (65.17)	
Middle level and above	543 (34.79)	218 (34.71)	325 (34.83)	
**Work years**				<**0.001**
≤ 10 years	733 (46.96)	255 (40.61)	478 (51.23)	
>10 years	828 (53.04)	373 (59.39)	455 (48.77)	
**Education level**				<**0.001**
Senior high school and below	348 (22.29)	172 (27.39)	176 (18.86)	
Bachelor degree or above	1,213 (77.71)	456 (72.61)	757 (81.14)	
**Political status**				**0.188**
Other	1,206 (77.26)	474 (75.48)	732 (78.46)	
Member of the Communist Party of China	355 (22.74)	154 (24.52)	201 (21.54)	
**Job location**				**0.023**
Rural	705 (45.16)	306 (48.73)	399 (42.77)	
Urban	856 (54.84)	322 (51.27)	534 (57.23)	
**Work environment**				<**0.001**
Mean (SD)	4.01 (0.87)	4.37 (0.66)	3.77 (0.90)	
**Remuneration**				<**0.001**
Mean (SD)	3.47 (1.11)	3.80 (1.01)	3.25 (1.11)	
**Work intensity**				<**0.001**
Mean (SD)	3.39 (1.06)	2.92 (1.09)	3.70 (0.92)	

### 3.1 Reliability and validity analysis

The overall Cronbach's α coefficient of the Chinese version of the MBI scale in this study was 0.859. The internal Cronbach's α coefficients of the dimensions of emotional exhaustion, depersonalization, and personal accomplishment were 0.926, 0.914, and 0.843, respectively. The split-half reliabilities of emotional exhaustion, depersonalization, and personal accomplishment were 0.869, 0.712, and 0.878, respectively.

The χ^2^ value of Bartlett's sphericity test was 20,333.90, *p* < 0.001. The Kaiser–Meyer–Olkin measure of sampling adequacy was 0.922. Three factors with the characteristic root λ > 1 were extracted by exploratory factor analysis, and the cumulative variance contribution rate was 73.01%. Factors 1, 2, and 3 explained the three dimensions of personal accomplishment, depersonalization, and emotional exhaustion in the MBI scale, respectively (Supplementary Table S1).

### 3.2 Factors affecting job burnout based on MBI

#### 3.2.1 Univariate analysis of factors affecting job burnout

The scores for the emotional exhaustion, depersonalization, and personal accomplishment dimensions of the PHCWs were 2.89 ± 1.38, 2.20 ± 1.34, and 3.74 ± 1.42 (see Supplementary Table S2).

The category scores measured by the MBI subscale were taken as the norm (28) and compared with our results. The mean scores for emotional exhaustion and depersonalization among Chinese PHCWs were higher than the general population norm and medical personnel norm; meanwhile, the mean scores for the personal accomplishment dimension were lower. All differences were statistically significant. This indicates that there is a high level of burnout among PHCWs in China (Supplementary Table S2).

The survey revealed that the burnout rate of PHCWs in China was 59.77% (933/1,561), among which 857 (54.90%) had mild burnout and 76 (4.87%) had severe burnout. Univariate statistical analysis revealed significant differences between burnout and non-burnout PHCWs for the following variables: age, work unit, years of work, education level, and work location (*p* < 0.05).

#### 3.2.2 Logistic analysis of factors affecting PHCW burnout

**Figure 1:**
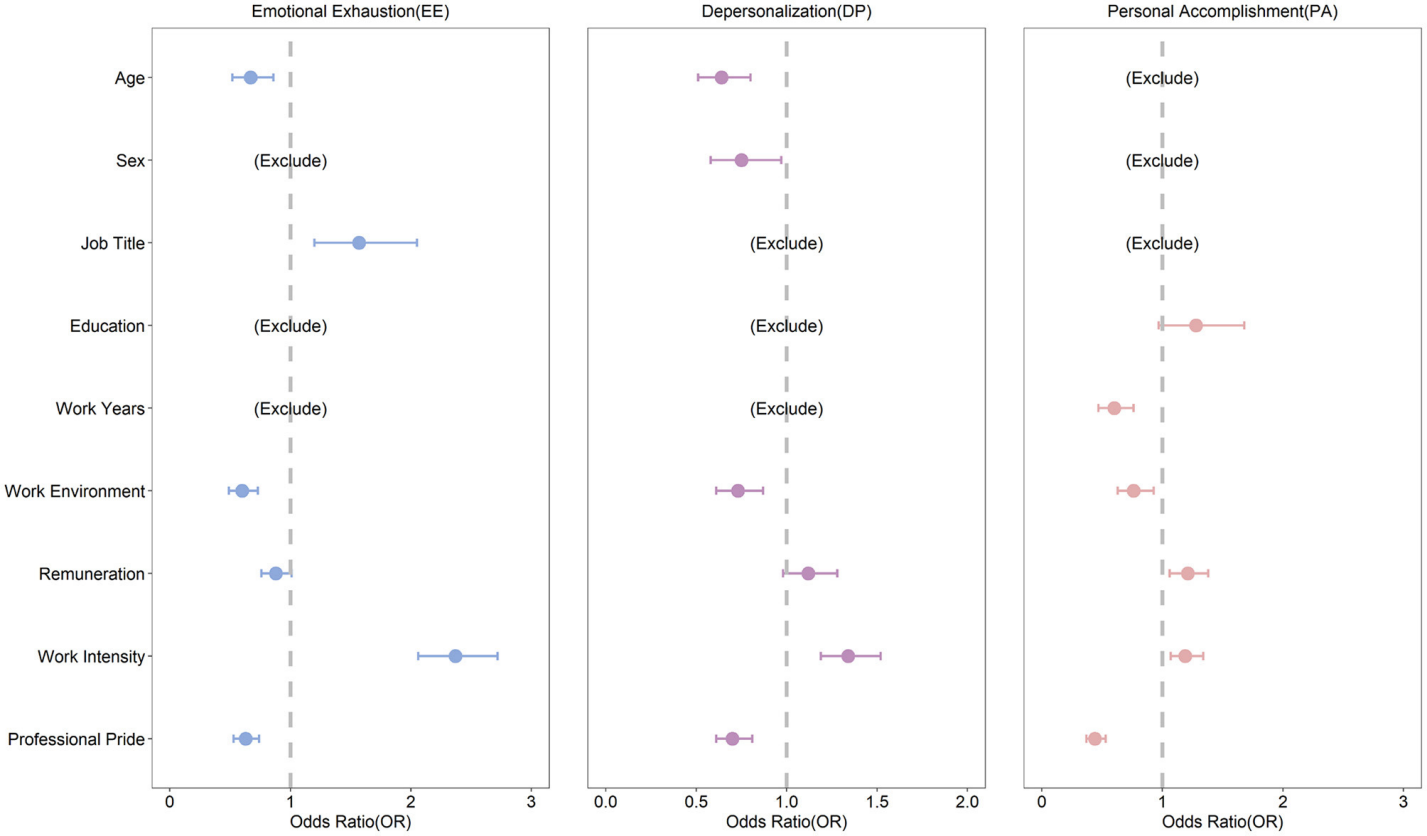
Forest plot of logistic analysis of burnout influencing factors.

We established a logistic regression model using the stepwise regression method. Sociodemographic factors, work environment, work treatment, work intensity, and professional pride were included as independent variables in the initial logistic regression. Taking the no-job-burnout group as the control group, we conducted regression analysis with emotional exhaustion, personality disintegration, and personal accomplishment burnout (mild job burnout + severe job burnout) as dependent variables. Figure 1 shows the results.

For the emotional exhaustion dimension, the results showed that PHCWs aged > 35 years had lower scores for emotional exhaustion (OR: 0.67; 95% CI: 0.52–0.86). Better work environment (OR: 0.60; 95% CI: 0.49–0.73) and higher professional pride (OR: 0.63; 95% CI: 0.53–0.74) were associated with lower scores for emotional exhaustion. Meanwhile, PHCWs with high work intensity (OR: 2.37; 95% CI: 2.06–2.72) and higher technical titles (OR: 1.57; 95% CI: 1.20–2.05) had higher scores for emotional exhaustion.

For the depersonalization dimension, PHCWs aged > 35 years had lower depersonalization scores compared with those aged ≤ 35 years (OR: 0.64; 95% CI: 0.51–0.80). Females had lower depersonalization scores compared with males (OR: 0.75; 95% CI: 0.58–0.97). Similar to the emotional exhaustion dimension, better work environment (OR: 0.73; 95% CI: 0.61–0.87) and higher professional pride (OR: 0.70; 95% CI: 0.61–0.81) were associated with lower scores for depersonalization while high work intensity (OR: 1.34; 95% CI: 1.19–1.52) was associated with higher scores.

For the personal accomplishment dimension, over 10 years of work experience (OR: 0.60; 95% CI: 0.47–0.76), better work environment (OR: 0.76; 95% CI: 0.63–0.93), and lower professional pride (OR: 0.44; 95% CI: 0.37–0.53) were significantly associated with lower scores for personal accomplishment. Higher remuneration (OR: 1.21; 95% CI: 1.06–1.38) and higher work intensity (OR: 1.19; 95% CI: 1.07–1.34) were significantly associated with higher scores for personal accomplishment.

#### 3.2.3 Structural equation model analysis

**Figure 2:**
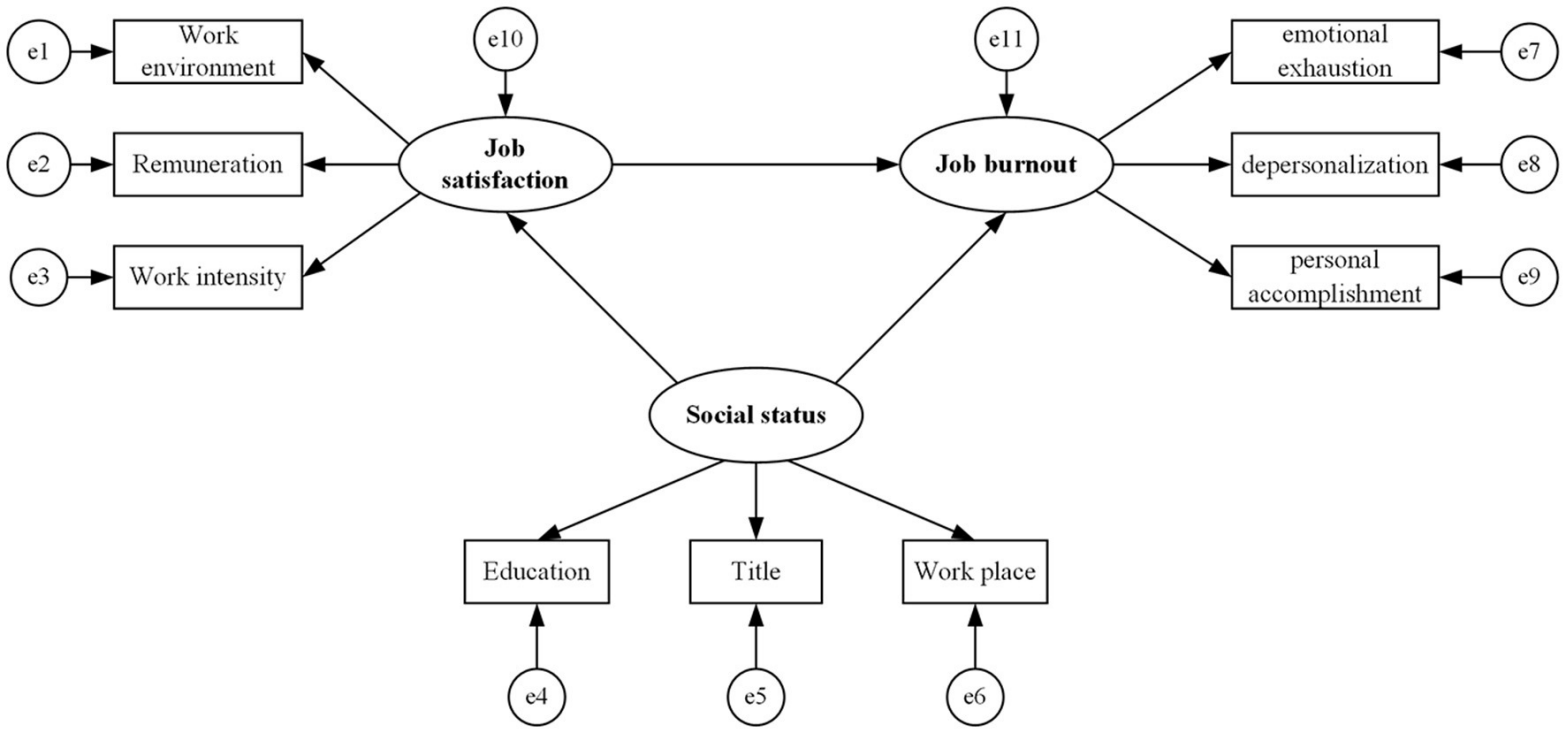
Structural equation model framework of job burnout.

Based on this study's theoretical hypothesis and the previous analysis of the factors affecting job burnout, SEM was constructed as shown in Figure 2. There were three observed variables of social status: education, technical title, and workplace; three observed variables of job satisfaction: work environment, salary, and work intensity; and three observed variables of burnout: emotional exhaustion, personality disintegration, and personal fulfillment.

The observed variable data were substituted into the SEM, and the model was fitted using the maximum likelihood method. The main fitting indexes of the model roughly reached the criteria for fitness, indicating acceptable model fit (Supplementary Table S3).

In the SEM, the standardized direct effect of job satisfaction on burnout was −0.352, that of social status on job satisfaction was −0.260, and that of social status on burnout was 0.165. All standardized direct effects were statistically significant. The regression coefficients of all observed variables of job satisfaction and social status reached statistical significance, indicating that each observed variable of the measurement model could explain the latent variables well.

We further used multigroup invariance modeling to explore the similarities and differences in the SEM between different groups to improve the empirical validity of the factors affecting burnout. We selected the sociodemographic variables of age, gender, years of work, and whether engaged in new COVID-19-related work for multigroup analysis.

**Figure 3:**
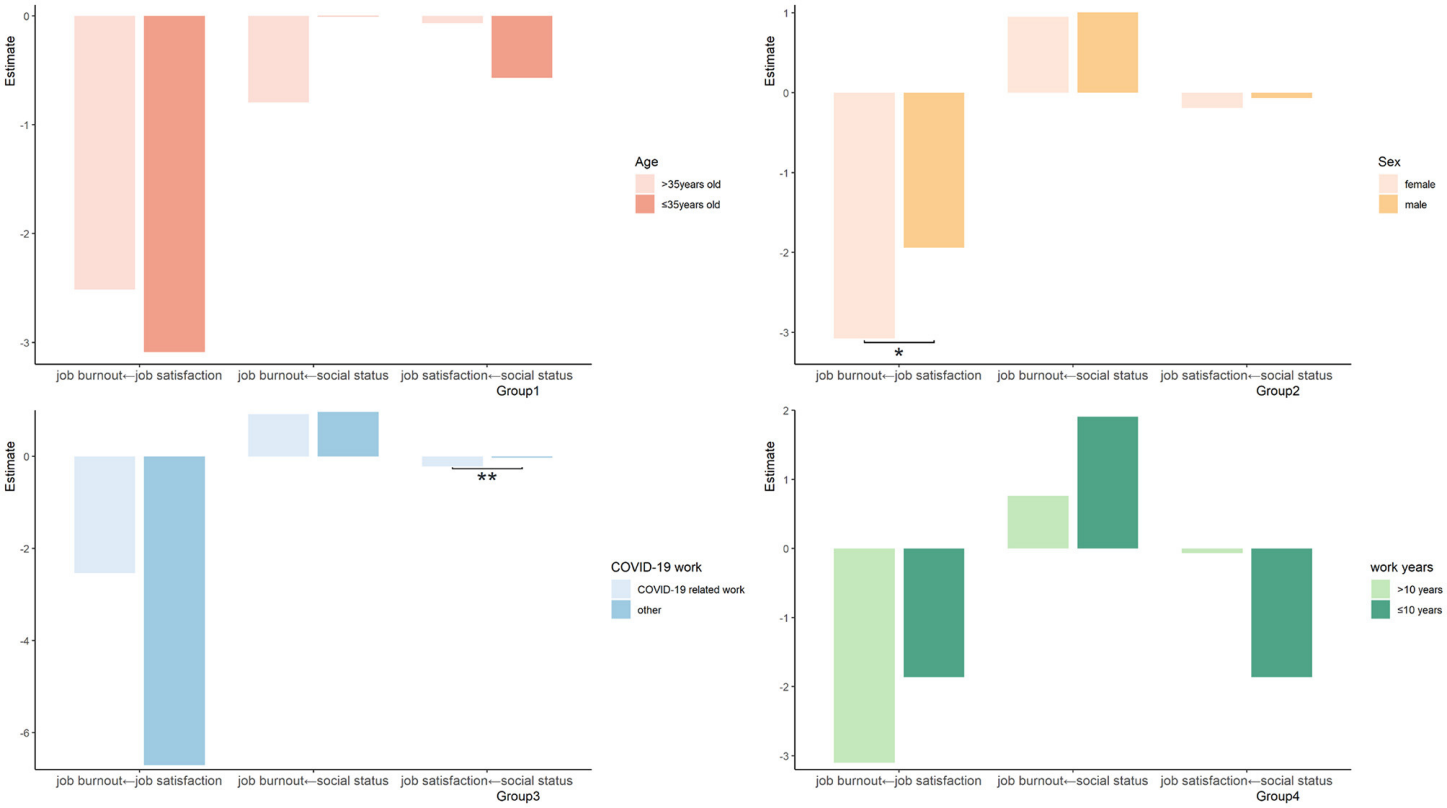
Multigroup invariance modeling of job burnout in different groups. *p < 0.05, **p < 0.01.

PHCWs were divided into a younger group (≤35 years) and elder group (>35 years), a male and female group, a COVID-19-related work group and others, and a short work experience group (≤10 years) and long work experience group (>10 years). When the absolute value of the critical ratio of the path coefficient difference between different groups is >1.96, the corresponding path coefficient difference between groups is significant; that is, *p* < 0.05. Multigroup analysis showed that the influence of job satisfaction on job burnout was more significant among females (−3.079 vs. −1.940). The effect of social status on job burnout was more significant for the COVID-19-related work group (−0.221 vs. −0.029). There was no significant difference in the path coefficients among other models (Figure 3).

### 3.3 Preferred ways to reduce job burnout

The option ranking method was used for the preferred ways to reduce job burnout. We presented five ways to reduce job burnout: (1) awarding an honorary certificate, (2) reducing work intensity, (3) improving the work environment, (4) providing opportunities for further study, and (5) Increasing wages.

**Figure 4:**
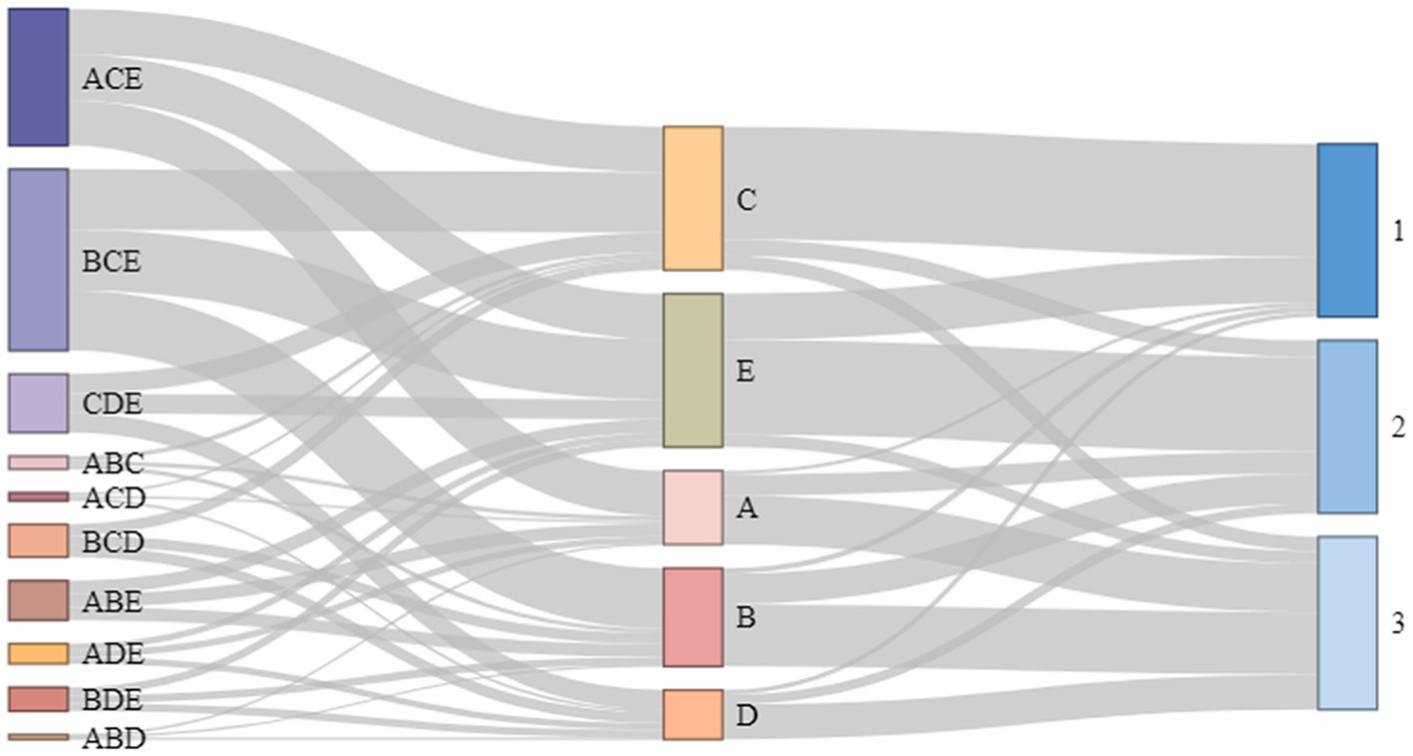
Sankey diagram of preference for improving job burnout. **(A)** Awarding an honorary certificate, **(B)** Improving work intensity, **(C)** Improving work environment, **(D)** Providing opportunities for further study, and **(E)** Increasing wages and allowances.

Increasing wages (88.68%) and improving the work environment (83.09%) were found to be most effective. Analyzing the combination of options, most healthcare workers (34.98%) reduced their work intensity, improved their work environment, and increased their salaries. According to the ranking analysis of the importance of the options, the most effective healthcare workers (64.95%) can improve the work environment (Figure 4).

Based on the Thurston method, the scale values of the five options (1, 2, 3, 4, and 5) were ranked on a psychological valence chart. The results showed that the most-preferred ways for PHCWs to reduce job burnout were improving the work environment (0.913) and increasing salaries (0.810). Meanwhile, the scale values of awarding honorary certificates or titles (−0.342), reducing work intensity (−0.594), and providing opportunities for further study (−0.787) were all negative (Supplementary Figure S1).

## 4 Discussion

### 4.1 Chinese version of the MBI scale has good reliability and validity

The MBI scale has been widely used to measure job burnout. During COVID-19, this scale was used in Italy, the US, Belgium, India, Singapore, and other countries to measure the job burnout of frontline healthcare workers, and its reliability and validity were verified (29–32).

We used a modified Chinese version of the MBI burnout scale to conduct a presurvey and test scale reliability and validity at 10 sites in Heilongjiang, Sichuan, Gansu, Anhui, and Shandong Provinces. The Cronbach's α coefficients for emotional exhaustion, depersonalization, and personal accomplishment were >0.7, demonstrating that the scale had good reliability, internal consistency, and external consistency.

### 4.2 During the COVID-19 pandemic, job burnout was common among PHCWs

The overall reported job burnout rate among PHCWs was about 60%; mild burnout accounted for 55%, and severe burnout accounted for 5%. Galanis summarized 16 studies where the MBI was used to measure nurses' job burnout and found that the emotional exhaustion rate was 34%, the personality disintegration rate was 12%, and the low personal achievement rate was 15% (33). Compared with previous findings, the job burnout rate among PHCWs in China was found to be significantly higher (34, 35), suggesting that this issue warrants attention in China.

The results suggested that job burnout among PHCWs is characterized by high emotional exhaustion, high personality disintegration, and low personal accomplishment, among which the score for personal accomplishment was significantly below the norm. This is similar to the findings of Hu et al. (36) and Parandeh et al. (37). It can be attributed to the fact that PHCWs in China were mostly engaged in repetitive tasks with high work pressure and long work hours during the pandemic (38) and may have encountered unsupportive or uncooperative patients (39).

PHCWs played an important role in the struggle to contain COVID-19 (40). From 2020 to 2022, China's approach to the pandemic shifted from “zero clearing” to “dynamic clearing” (41, 42). PHCWs took on the tedious work of COVID-19 patient treatment, nucleic acid testing, epidemiological investigation, vaccination, isolation and prevention, and decontamination (43, 44), which involved long work hours and high work pressure.

### 4.3 Factors affecting burnout among PHCWs in China

The factors affecting burnout were diverse and changed over time, and could be both subjective and objective. Our findings showed that burnout level was associated with the age, years of work, education level, and work location of healthcare workers. Among them, age ≥ 35 years, better work environments, and more professional pride inhibited emotional exhaustion while more intense work exacerbated it. Gambaro et al.'s study on job burnout of healthcare workers also showed a negative correlation between age and job burnout. Similar to our findings, work experience has been shown to supply healthcare workers with the knowledge and emotion regulation skills they need to mitigate burnout (45). During the COVID-19 pandemic, healthcare staff with higher titles often had to assume more responsibilities, thus leading to emotional exhaustion (8, 46).

The depersonalization dimension was similar to that of emotional exhaustion. Therefore, PHCWs who are younger, have higher technical titles, and have higher work intensity should be the focus of interventions for burnout. In addition, females were less likely to show symptoms of depersonalization (i.e., holding negative or inappropriate attitudes toward their work objects) (47). Previous studies have also shown that female healthcare workers have more empathy for patients, better understand patients, and devote more time to them (48–50).

In the personal accomplishment dimension, healthcare workers with more than 10 years of work experience had lower levels of personal fulfillment. Studies have shown that longer years of work are usually a contributing factor to burnout (51, 52). Different from previous studies, we found that those with high work intensity showed a higher level of personal accomplishment (36). This reflects the sense of social responsibility and dedication shown by Chinese healthcare workers during the pandemic (38).

Our results highlight the important role of job satisfaction in reducing healthcare workers' job burnout. SEM showed that improving job satisfaction could reduce job burnout. Our findings partly confirm Goulet's and Singh theory of career commitment—that is, job satisfaction has a negative effect on job burnout (53). Therefore, as an important factor affecting PHCWs' professional development, job satisfaction should be an important intervention strategy in occupational health (54). Social status also affects the job satisfaction of healthcare workers, thus affecting job burnout. Thus, more attention should be paid to groups with higher social status (38).

### 4.4 Burnout improvement preferences of PHCWs in China

Among the ways to reduce the job burnout among PHCWs, improving the work environment and increasing wages are the most important. During the COVID-19 pandemic, healthcare workers often worked in isolation wards and temporary nucleic acid test sites, and the work environments were relatively harsh, which could easily lead to burnout (38). Healthcare workers expressed the most dissatisfaction with the remuneration dimension. PHCWs in China have low salaries but bear higher workloads, greater risks of infection, and heavier physical and mental pressure (8, 55, 56). Incentive policies should be implemented to improve healthcare workers' job satisfaction and alleviate burnout by increasing their income.

## 5 Strength and limitations

This study used a cross-sectional survey that only reflected burnout levels at the time of the survey. Preexisting psychopathological conditions should be taken into consideration. It would be beneficial to confirm causality with longitudinal data in future studies. Second, we used the revised MBI scale to measure job burnout. Although the scale has good reliability and validity, it might be slightly different from the norm, which reduces comparability to some extent. Finally, we used multicenter convenience sampling. Although the survey area was selected in consideration of economic and geographic location and balanced urban/rural distribution, it did not strictly follow random sampling for the whole country, and the sample had large gender differences. Our research was based on the respondents and did not collect the characteristics of non-respondents. Therefore, the conclusions only represent the respondents, which might lead to non-response bias and underestimate or overestimate the level of job burnout. Thus, caution should be exercised in extrapolating from the conclusions.

## 6 Conclusion

We found that PHCWs in China had high levels of job burnout during the COVID-19 pandemic. Job burnout among PHCWs was related to their age, years of work, education level, and workplace and was influenced by job satisfaction and professional identity. At present, PHCWs in China have average salaries but high work intensity. Improving their work environments and salaries could reduce their job burnout. Healthcare managers can refer to healthcare workers' preferred ways to reduce job burnout and provide support to maintain their work enthusiasm and thus the stability of the whole healthcare system.

We evaluated the level of job burnout among PHCWs in China during COVID-19, analyzed its influencing factors, and summarized the preferred ways to reduce job burnout. However, this study is a cross-sectional study with a risk of non-response bias. Further evaluation is needed to inform future practice.

## Data availability statement

The datasets generated and/or analysed during the current study are available from the corresponding author on reasonable request.

## Ethics statement

This study was approved by the Ethics Committee of Peking University Health Science Center, China (approval number: IRB00001052-21132) and the signal-free informed consent application was approved.

## Author contributions

XC: Data curation, Formal analysis, Investigation, Methodology, Software, Visualization, Writing – original draft. TZ: Data curation, Formal analysis, Investigation, Methodology, Software, Visualization, Writing – original draft, Writing – review & editing. LC: Investigation, Methodology, Validation, Writing – review & editing. SZ: Methodology, Writing – review & editing, Formal analysis. AY: Investigation, Supervision, Writing – review & editing. XS: Investigation, Supervision, Writing – review & editing. SG: Investigation, Supervision, Writing – review & editing. YZ: Investigation, Supervision, Writing – review & editing. CW: Investigation, Supervision, Writing – review & editing. JD: Investigation, Project administration, Supervision, Validation, Writing – review & editing. YL: Investigation, Project administration, Supervision, Validation, Writing – review & editing. Q-BL: Investigation, Project administration, Supervision, Validation, Writing – review & editing. FC: Data curation, Funding acquisition, Investigation, Methodology, Project administration, Resources, Supervision, Validation, Writing – review & editing.

## References

[1] Thompson SL Salmon JW. Strikes by physicians: a historical perspective toward an ethical evaluation. Int J Health Serv. (2006) 36:331–54. 10.2190/B5CX-UX69-45LY-2D6D 16878396

[2] Robinson SE Roth SL Keim J Levenson M Flentje JR Bashor K. Nurse burnout: work related and demographic factors as culprits. Res Nurs Health. (1991) 14:223–8. 10.1002/nur.4770140309 1887102

[3] Parker PA Kulik JA. Burnout, self- and supervisor-rated job performance, and absenteeism among nurses. J Behav Med. (1995) 18:581–99. 10.1007/BF01857897 8749987

[4] Lee RT Ashforth BE A. meta-analytic examination of the correlates of the three dimensions of job burnout. J Appl Psychol. (1996) 81:123–33. 10.1037/0021-9010.81.2.123 8603909

[5] Vahey DC Aiken LH Sloane DM Clarke SP Vargas D. Nurse burnout and patient satisfaction. Med Care. (2004) 42:Ii57–66. 10.1097/01.mlr.0000109126.50398.5a 14734943 PMC2904602

[6] Barck-Holst P Nilsonne Å Åkerstedt T Hellgren C. Coping with stressful situations in social work before and after reduced working hours, a mixed-methods study. Eur J Soc Work. (2021) 24:94–108. 10.1080/13691457.2019.1656171

[7] Khodadadi A Ravari A Sayadi A khodadadi H Jafarinaveh H. Occupational burnout assessment among nurses working in Iranian hospital of Ali-ebn Abitaleb, Rafsanjan- Iran. J Occup Health Epidemiol. (2012) 1:103–10. 10.18869/acadpub.johe.1.2.103

[8] Zhang LF You LM Liu K Zheng J Fang J-b Lu M. The association of Chinese hospital work environment with nurse burnout, job satisfaction, and intention to leave. Nurs Outlook. (2014) 62:128–37. 10.1016/j.outlook.2013.10.010 24345617 PMC3959248

[9] Li X Lu J Hu S Cheng KK Maeseneer J Meng Q. The primary health-care system in China. Lancet. (2017) 390:2584–94. 10.1016/S0140-6736(17)33109-4 29231837

[10] Wang H Jin Y Wang D Zhao S Sang X Yuan B. Job satisfaction, burnout, and turnover intention among primary care providers in rural China: results from structural equation modeling. BMC Fam Pract. (2020) 21:12. 10.1186/s12875-020-1083-8 31941455 PMC6961377

[11] Scanlan JN Still M. Job satisfaction, burnout and turnover intention in occupational therapists working in mental health. Aust Occup Ther J. (2013) 60:310–8. 10.1111/1440-1630.12067 24089982

[12] West CP Tan AD Habermann TM Sloan JA Shanafelt TD. Association of resident fatigue and distress with perceived medical errors. Jama. (2009) 302:1294–300. 10.1001/jama.2009.1389 19773564

[13] Shanafelt TD Balch CM Bechamps G Russell T Dyrbye L Satele D. Burnout and medical errors among American surgeons. Ann Surg. (2010) 251:995–1000. 10.1097/SLA.0b013e3181bfdab3 19934755

[14] Dyrbye LN Shanafelt TD. Physician burnout: a potential threat to successful health care reform. JAMA. (2011) 305:2009–10. 10.1001/jama.2011.652 21586718

[15] Shanafelt TD Balch CM Dyrbye L Bechamps G Russell T Satele D. Special report: suicidal ideation among American surgeons. Arch Surg. (2011) 146:54–62. 10.1001/archsurg.2010.292 21242446

[16] West CP Tan AD Shanafelt TD. Association of resident fatigue and distress with occupational blood and body fluid exposures and motor vehicle incidents. Mayo Clin Proc. (2012) 87:1138–44. 10.1016/j.mayocp.2012.07.021 23218084 PMC3541922

[17] Al-Dubai SAR Ganasegeran K Perianayagam W Rampal KG. Emotional burnout, perceived sources of job stress, professional fulfillment, and engagement among medical residents in Malaysia. Sci World J. (2013) 2013:137620. 10.1155/2013/137620 24367238 PMC3842044

[18] Al-Dubai SA Rampal KG. Prevalence and associated factors of burnout among doctors in Yemen. J Occup Health. (2010) 52:58–65. 10.1539/joh.O8030 19907108

[19] Siu C Yuen SK Cheung A. Burnout among public doctors in Hong Kong: cross-sectional survey. Hong Kong Med J. (2012) 18:186–92. 22665681

[20] Edwards D Burnard P Coyle D Fothergill A Hannigan B A. stepwise multivariate analysis of factors that contribute to stress for mental health nurses working in the community. J Adv Nurs. (2001) 36:805–13. 10.1046/j.1365-2648.2001.02035.x 11903710

[21] Bank TW. Population of China [EB/OL]. (2021). Available online at: https://data.worldbank.org/country/china (accessed May 7, 2023).

[22] Bank TW. World Development Indicators of China [EB/OL]. (2021). Available online at: https://databank.worldbank.org/reports.aspx?source=2&country=CHN (accessed May 7, 2023).

[23] Lo D Wu F Chan M Chu R Li D. A systematic review of burnout among doctors in China: a cultural perspective. Asia Pac Fam Med. (2018) 17:3. 10.1186/s12930-018-0040-3 29449785 PMC5806482

[24] Yao H Wang P Tang Y-L Liu Y Liu T Liu H. Burnout and job satisfaction of psychiatrists in China: a nationwide survey. BMC Psychiatry. (2021) 21:593. 10.1186/s12888-021-03568-6 34819029 PMC8612106

[25] Maslach C Jackson SE. The measurement of experienced burnout. J Organ Behav. (1981) 2:99–113. 10.1002/job.4030020205

[26] Ahola K Gould R Virtanen M Honkonen T Aromaa A Lönnqvist J. Occupational burnout as a predictor of disability pension: a population-based cohort study. Occup Environ Med. (2009) 66:284–90; discussion 2–3. 10.1136/oem.2008.038935 19017706

[27] Kalimo R Pahkin K Mutanen P Topipinen-Tanner S. Staying well or burning out at work: work characteristics and personal resources as long-term predictors. Workand Stress. (2003) 17:109–22. 10.1080/0267837031000149919

[28] Maslach CJSE Leiter MP. Maslach burnout inventory manual. Palo Alto, VA: Consulting Psychologists Press (1996).

[29] Lasalvia A Amaddeo F Porru S Carta A Tardivo S Bovo C. Levels of burn-out among healthcare workers during the COVID-19 pandemic and their associated factors: a cross-sectional study in a tertiary hospital of a highly burdened area of north-east Italy. BMJ Open. (2021) 11:e045127. 10.1136/bmjopen-2020-045127 33455940 PMC7813385

[30] Lasater KB Aiken LH Sloane DM French R Martin B Reneau K. Chronic hospital nurse understaffing meets COVID-19: an observational study. BMJ Qual Saf. (2021) 30:639–47. 10.1136/bmjqs-2020-011512 32817399 PMC7443196

[31] Bruyneel A Smith P Tack J Pirson M. Prevalence of burnout risk and factors associated with burnout risk among ICU nurses during the COVID-19 outbreak in French speaking Belgium. Intens Crit Care Nurs. (2021) 65:103059. 10.1016/j.iccn.2021.103059 33875341 PMC9759739

[32] Jose S Dhandapani M Cyriac MC. Burnout and resilience among frontline nurses during COVID-19 pandemic: a cross-sectional study in the emergency department of a tertiary care center, North India. Indian J Crit Care Med. (2020) 24:1081–8. 10.5005/jp-journals-10071-23667 33384515 PMC7751034

[33] Galanis P Vraka I Fragkou D Bilali A Kaitelidou D. Nurses' burnout and associated risk factors during the COVID-19 pandemic: a systematic review and meta-analysis. J Adv Nurs. (2021) 77:3286–302. 10.1111/jan.14839 33764561 PMC8250618

[34] Wang Z Xie Z Dai J Zhang L Huang Y Chen B. Physician burnout and its associated factors: a cross-sectional study in Shanghai. J Occup Health. (2014) 56:73–83. 10.1539/joh.13-0108-OA 24430838

[35] Low ZX Yeo KA Sharma VK Leung GK McIntyre RS Guerrero A. Prevalence of burnout in medical and surgical residents: a meta-analysis. Int J Environ Res Public Health. (2019) 16:1479. 10.3390/ijerph16091479 31027333 PMC6539366

[36] Hu D Kong Y Li W Han Q Zhang X Zhu LX. Frontline nurses' burnout, anxiety, depression, and fear statuses and their associated factors during the COVID-19 outbreak in Wuhan, China: a large-scale cross-sectional study. EClinicalMedicine. (2020) 24:100424. 10.1016/j.eclinm.2020.100424 32766539 PMC7320259

[37] Parandeh A Ashtari S Rahimi-Bashar F. Prevalence of burnout among health care workers during coronavirus disease (COVID-19) pandemic: a systematic review and meta-analysis. Prof Psychol Res Pract. (2022) 53:564–73. 10.1037/pro0000483

[38] Wan Z Lian M Ma H Cai Z Xianyu Y. Factors associated with burnout among Chinese nurses during COVID-19 epidemic: a cross-sectional study. BMC Nurs. (2022) 21:51. 10.1186/s12912-022-00831-3 35227272 PMC8883459

[39] Sun H Zhao Y. Analysis and consideration on the current situation of resource allocation of licensedregistered nurses in China(in Chinese). Chinese Hospitals. (2019) 23:42–5.

[40] Haldane V De Foo C Abdalla SM Jung AS Tan M Wu S. Health systems resilience in managing the COVID-19 pandemic: lessons from 28 countries. Nat Med. (2021) 27:964–80. 10.1038/s41591-021-01381-y 34002090

[41] Liu J Liu M Liang W. The dynamic COVID-zero strategy in China. China CDC Wkly. (2022) 4:74–5. 10.46234/ccdcw2022.015 35186372 PMC8837441

[42] Li Z Chen Q Feng L Rodewald L Xia Y Yu H. Active case finding with case management: the key to tackling the COVID-19 pandemic. Lancet. (2020) 396:63–70. 10.1016/S0140-6736(20)31278-2 32505220 PMC7272157

[43] Ministry of Commerce Peoples's Republic of China. The Joint Prevention and Control Mechanism of the State Council Press Conference Text 2020.05.16 [EB/OL]. (2020). Available online at: http://www.nhc.gov.cn/xcs/fkdt/202005/e78dfc196504497586f324f0d9a5bc36.shtml (accessed March 30, 2023).

[44] Li Q Liu W Wang J-Y Wang X-G Hao B Hu Y-B. Prevalence and risk factors of post-traumatic stress disorder symptoms among Chinese health care workers following the COVID-19 pandemic. Heliyon. (2023) 9:e14415. 10.1016/j.heliyon.2023.e14415 36974320 PMC9998286

[45] Gambaro E Gramaglia C Marangon D Azzolina D Probo M Rudoni M. The mediating role of gender, age, COVID-19 symptoms and changing of mansion on the mental health of healthcare workers operating in italy during the first wave of the COVID-19 pandemic. Int J Environ Res Public Health. (2021) 18:13083. 10.3390/ijerph182413083 34948696 PMC8700931

[46] Xie J Li J Wang S Li L Wang K Duan Y. Job burnout and its influencing factors among newly graduated nurses: a cross-sectional study. J Clin Nurs. (2021) 30:508–17. 10.1111/jocn.15567 33205476

[47] Finstad GL Giorgi G Lulli LG Pandolfi C Foti G León-Perez JM. Resilience, coping strategies and posttraumatic growth in the workplace following COVID-19: a narrative review on the positive aspects of trauma. Int J Environ Res Public Health. (2021) 18:9453. 10.3390/ijerph18189453 34574378 PMC8468098

[48] Fukui S Wu W Salyers MP. Impact of supervisory support on turnover intention: the mediating role of burnout and job satisfaction in a longitudinal study. Adm Policy Ment Health. (2019) 46:488–97. 10.1007/s10488-019-00927-0 30810850

[49] Cho E Jeon S. The role of empathy and psychological need satisfaction in pharmacy students' burnout and well-being. BMC Med Educ. (2019) 19:43. 10.1186/s12909-019-1477-2 30717723 PMC6360713

[50] Barnett MD Martin KJ Garza CJ. Satisfaction with work-family balance mediates the relationship between workplace social support and depression among hospice nurses. J Nurs Scholarsh. (2019) 51:187–94. 10.1111/jnu.12451 30570211

[51] Pradas-Hernández L Ariza T Gómez-Urquiza JL Albendín-García L De la Fuente EI Cañadas-De la Fuente GA. Prevalence of burnout in paediatric nurses: a systematic review and meta-analysis. PLoS ONE. (2018) 13:e0195039. 10.1371/journal.pone.0195039 29694375 PMC5918642

[52] Shiao JS Koh D Lo LH Lim MK Guo YL. Factors predicting nurses' consideration of leaving their job during the SARS outbreak. Nurs Ethics. (2007) 14:5–17. 10.1177/0969733007071350 17334166

[53] Goulet LR Singh P. Career commitment: a reexamination and an extension. J Vocat Behav. (2002) 61:73–91. 10.1006/jvbe.2001.1844

[54] Chen H Liu F Pang L Liu F Fang T Wen Y. Are you tired of working amid the pandemic? The role of professional identity and job satisfaction against job burnout. Int J Environ Res Public Health. (2020) 17:9188. 10.3390/ijerph17249188 33316964 PMC7764790

[55] Shi X Xiong D Zhang X Han M Liu L Wang J. Analysis of factors influencing the job satisfaction of medical staff in tertiary public hospitals, China: A cross-sectional study. Front Psychol. (2023) 14:1048146. 10.3389/fpsyg.2023.1048146 36818068 PMC9932040

[56] Wu H Liu L Wang Y Gao F Zhao X Wang L. Factors associated with burnout among Chinese hospital doctors: a cross-sectional study. BMC Public Health. (2013) 13:786. 10.1186/1471-2458-13-786 23985038 PMC3765838

