# Supplementary material for: Job burnout among primary healthcare workers during COVID-19 pandemic: cross-sectional study in China
Source: Front Public Health. 2023 Dec 6;11:1266864. doi: 10.3389/fpubh.2023.1266864 (PMC10731250; doi:10.3389/fpubh.2023.1266864)
Supplement: Supplementary file 1 [file Data_Sheet_1.pdf]

Table S1 Factor loadings and Variance Contribution Rate of the MBI Scale

**Factor**	**Dimension**	**Entry**	**Factor loadings**	**Eigenvalue**	**Variance Contribution Rate (%)**	**Cumulative Variance Contribution Rate (%)**
Factor 1	Personal accomplishment	personal accomplishment 1	0.578	6.400	37.649	37.649
		personal accomplishment2	0.805			
		personal accomplishment3	0.789			
		personal accomplishment4	0.865			
		personal accomplishment5	0.864			
		personal accomplishment6	0.898			
		personal accomplishment7	0.848			
Factor 2	Depersonalization	depersonalization1	0.806	4.684	27.552	65.201
		depersonalization2	0.839			
		depersonalization3	0.833			
Factor 3	Emotional exhaustion	emotional exhaustion1	0.784	1.328	7.811	73.012
		emotional exhaustion2	0.863			
		emotional exhaustion3	0.808			
		emotional exhaustion4	0.430			
		emotional exhaustion5	0.693			
		emotional exhaustion6	0.484			
		emotional exhaustion7	0.344			

Table S2 Score of MBI scale among primary healthcare workers in China

MBI scale	Score, mean (SD)	General population norm (n=11067)		Medical personnel norm (n=1104)	
		mean	P	mean	P
Emotional exhaustion	2.89 (1.38)	2.33	＜0.001	2.47	＜0.001
Depersonalization	2.20 (1.34)	1.74	＜0.001	1.42	＜0.001
personal Accomplishment *	3.74 (1.42)	4.33	＜0.001	4.56	＜0.001

* negative score

Table S3 The structural equation model mainly fit index

	CFI	GFI	AGFI	SRMR	RMSEA	AIC
Model fit	0.910	0.978	0.958	0.012	0.063	213.12
Criteria for fitness	>0.9 better，>0.8 acceptable	>0.9 acceptable	>0.9 better，>0.8 acceptable	＜0.05better，＜0.08 acceptable	<0.08 better	the smaller，the better

Figure S1 Thurston method analysis of preference for improving job burnout

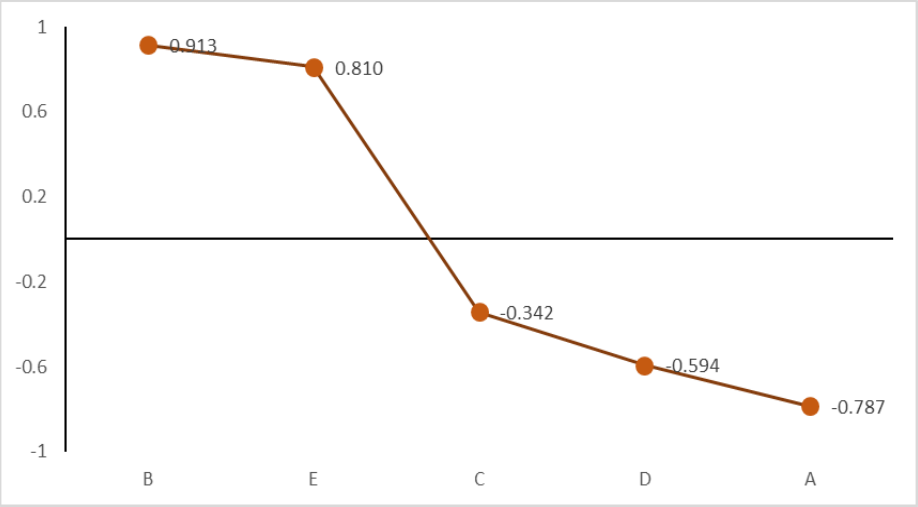

(A) Awarding an honorary certificate, (B) Improving work intensity, (C) Improving work environment, (D) Providing opportunities for further study, and (E) Increasing wages and allowances.
